# P-1856. Difference in T-cell responses to influenza internal proteins according to HLA type

**DOI:** 10.1093/ofid/ofae631.2017

**Published:** 2025-01-29

**Authors:** Yoon Kyung Cho, Hyun Mi Kang, Kyu Ri Kang, Jin Han Kang

**Affiliations:** The Catholic University of Korea, Seoul St. Mary's Hospital, Seoul, Seoul-t'ukpyolsi, Republic of Korea; The Catholic University of Korea, Seoul St. Mary's Hospital, Seoul, Seoul-t'ukpyolsi, Republic of Korea; Vaccine Bio Research Institute, Seoul, Seoul-t'ukpyolsi, Republic of Korea; The Catholic University of Korea, Seoul, Seoul-t'ukpyolsi, Republic of Korea

## Abstract

**Background:**

Gene variants in HLA are known to influence the degree of immune responses to influenza vaccines leading to varying immunogenicity among different populations. Universal influenza vaccines that contain internal proteins which elicit immunogenicity to various influenza strains are under development, and in order to provide the best protection for vaccine recipients, this study aimed to investigate difference in T-cell responses to influenza internal proteins according to HLA type.

**Methods:**

In the murine challenge model, HLA-A*02:01 and HLA-A*11:01 transgenic mice were immunized with a seasonal influenza vaccine and then injected intramuscularly with two doses of one the following internal proteins: nucleoprotein (NP), nonstructural protein 1 (NS1), nonstructural protein 2 (NS2), matrix protein 1 (M1), matrix protein 2 (M2), and polymerase acidic protein (PA)). The mice were then challenged with A/Cambodia/e0826360/2020 (H3N2) virus.

**Results:**

The NS1NS2 protein showed 0% survival in the A*11:01 type but a high immune response in the HLA-A*02:01 type. In the chemokine/cytokine analyses, the PA_A2.1 group followed by NS1NS2_A2.1, M1M2_A2.1, and M1M2_A11.1 showed the highest positive scores for cytokines MIP-1a, RANTES, IFN-γ, KC, and MCP-1. Furthermore, the PA_A11.1 group showed statistically valid IFN-γ and CD8+ T cell percentages in the lungs, whereas in the spleen, the PA_A2.1 group showed high percentages.

**Conclusion:**

Internal proteins that showed adequate immune responses in HLA-A*02:01 type was NS1NS2, and M1M2 showed adequate responses in both HLA-A*02:01 and HLA-A*11:01 type.

**Disclosures:**

All Authors: No reported disclosures

